# RETRACTION: Wound Healing Potential and In Silico Appraisal of *Convolvulus arvensis* L. Methanolic Extract

**DOI:** 10.1155/bmri/9868063

**Published:** 2025-10-28

**Authors:** BioMed Research International

RETRACTION: U. Saleem, S. Khalid, S. Zaib, et al., “Wound Healing Potential and In Silico Appraisal of *Convolvulus arvensis* L. Methanolic Extract,” *BioMed Research International* 2022 (2022): 1373160, https://doi.org/10.1155/2022/1373160.

The above article, published online on 24 November 2022 in Wiley Online Library (http://wileyonlinelibrary.com), has been retracted by agreement between the journal’s Section Editor Jinsong Ren; and John Wiley & Sons Ltd.

The retraction has been agreed following an investigation of the concerns raised by *Mycosphaerella arachidis* on PubPeer [1], which identified unexpected similarities between multiple panels of Figure 1.

More specifically, the image depicting the wound of a rat in the 10% ointment group after 8 days appears identical to the image of the animal in the 20% ointment group after 12 days.

As a result of the investigation, the data and conclusions of this article are considered unreliable.

Muhammad Furqan Akhtar and Ammara Saleem and disagree with this retraction. The other authors have been informed of this decision but did not provide a response.
